# Managing nirmatrelvir/ritonavir during COVID-19: pharmacists’ experiences from the Perak state of Malaysia

**DOI:** 10.1186/s40545-022-00469-1

**Published:** 2022-10-23

**Authors:** Chee Tao Chang, Su Yin Ong, Xin Jie Lim, Lan Sim Chew, Philip Rajan

**Affiliations:** 1 Clinical Research Centre, Hospital Raja Permaisuri Bainun, Ministry of Health Malaysia, Ipoh, Malaysia; 2 School of Pharmacy, Monash University Malaysia, Subang Jaya, Malaysia; 3 Perak Pharmaceutical Services Division, Ministry of Health Malaysia, Tanjung Rambutan, Malaysia; 4 Pharmacy Department, Hospital Raja Permaisuri Bainun, Ministry of Health Malaysia, Ipoh, Malaysia

## Abstract

Novel therapeutic agents for SARS-CoV-2 have emerged over time, serving to reduce the severity of the disease, admission and mortality, especially among high-risk populations. Oral nirmatrelvir/ritonavir (Paxlovid^®^) was found to reduce the risk of disease progression. Pharmacists played multiple roles in handling the COVID-19 pandemic. This article highlights the roles of pharmacists in managing nirmatrelvir/ritonavir within the Malaysian context. Pharmacists were actively involved in Paxlovid^®^ inventory management. To ensure the balance between supply and demand of new therapeutic drugs, pharmacists in health facilities constantly monitor the inventory levels of the medications. As Paxlovid^®^ was initially reserved for a certain population who met the clinical eligibility criteria based on a scoring system, pharmacists were required to screen and exclude patients with non-indications or contraindications to the medication. During dispensing, pharmacists convey clear instructions on how to take the medications to ensure adherence and medication safety. The novel nature of the medications necessitates pharmacists to counsel patients regarding its indication, the mode of action, actions to take when missing a dose or overdose happens, side effects, storage and disposal methods, as well as mechanism of reporting adverse drug reactions. Pharmacists were required to follow-up all patients via phone call on Day 3 and Day 5 post-initiation, examining both adherence and adverse drug reactions associated with Paxlovid^®^. Pharmacists experienced multiple challenges in managing Paxlovid^®^, particularly due to increased workload, suboptimal follow-up response, stringent medication storage requirements, and adherence issues. Universal research and innovation initiatives were proposed to improve the delivery of novel therapeutic agents in the future health system.

## Introduction

The SARS-CoV-2 Omicron subvariants BA.2.12.1 and BA.4/5 have shown a sharp rise. The effectiveness of COVID-19 vaccines and therapeutic monoclonals may be jeopardized by these new subvariants, which carry further mutations in their spike proteins. BA.4/5 is 4.2-fold more resistant, and as a result, more likely to cause vaccine breakthrough infections [1]. Meanwhile, novel therapeutic agents have emerged over time, serving to reduce the severity of the disease, admission and mortality, especially among high-risk populations. Oral nirmatrelvir/ritonavir (Paxlovid^®^) combination, a protease inhibitor, was introduced in early 2022. It was reported that this combination could reduce the risk of disease progression significantly by 89%, with a considerably safe profile [2].

Pharmacists played multiple roles in handling the COVID-19 pandemic, including innovating medication delivery and monitoring systems; inventory management and procurement; development of treatment guidelines; supporting clinical trials; and ensuring the continuity of pharmaceutical care and counselling [3–6]. The roles of pharmacists in handling novel drugs have not been reported previously. This article highlights the roles of pharmacists, challenges, and potential innovations in managing Paxlovid^®^, a novel therapeutic agent within the Malaysian context.

### Inventory management and distribution of medicines within the hospital

The Perak state consists of five publicly funded tertiary hospitals with specialists, 11 secondary hospitals without specialists, and 11 district health offices. Pharmacists’ leadership was crucial in the face of drug shortages during COVID-19 [7]. To ensure the balance between supply and demand for Paxlovid^®^, pharmacists in these facilities were tasked to constantly monitor the inventory levels of Paxlovid^®^. Online spreadsheets were used to collect and monitor the usage of Paxlovid^®^ across different facilities. Pharmacists at the ground level were required to fill up the electronic inventory spreadsheet, which detailed in–out movement and the current stock level of Paxlovid^®^ on a daily basis. From the central level, pharmacists at the State Health Division closely monitored Paxlovid^®^ levels and activated an inventory mobilization plan, which involved the transit of Paxlovid^®^ from the major hospitals to the health district offices once the pre-determined minimum stock level was triggered. Meanwhile, liaison pharmacists in these facilities were required to update the patient administration registry and return medication registry, comprising the age, ethnicity, COVID-19 category, regimen, as well as the start and end date of therapy on a weekly basis.

In the initial stage, Paxlovid^®^ was available for patients who visited publicly funded hospitals and health clinics. Towards the end of June 2022, the supply of Paxlovid^®^ has been expanded to benefit patients who seek treatment from private facilities, including private hospitals and general practitioners [8]. Once the letter of intent and application form were received, the liaison pharmacists in charge of inventory management from public health clinics were required to mobilize inventory to the private health facilities. During the transportation of Paxlovid^®^, pharmacists were required to maintain the temperature between 15 and 25 °C to ensure stability [9]. Simultaneously, site pharmacists filled up the electronic Paxlovid^®^ inventory spreadsheet, which was subsequently reported to the Pharmacy State Health Division.

### Screening of patients

Screening of patients is routinely performed by pharmacists [10]. Paxlovid^®^ was reserved for a certain population who met the clinical eligibility criteria based on a set of scoring systems [11]. It was developed through reviewing of current literature and consensus among clinicians, including family medicine specialists, infectious disease specialists, and medical specialists [12]. Briefly, patients who were symptomatic for less than 5 days with a clinical stage of 2 or 3 were considered potential candidates for Paxlovid^®^ [7]. This population of patients was further stratified based on age, immunity, comorbidities, obesity, smoking, as well as vaccination status. Older adults, immunocompromised patients, patients with multiple comorbidities, obese and smoking patients, and those who did not complete COVID-19 vaccination were prioritized, given the fact that this population inherited a greater risk of progressing to severe COVID-19 stages [11, 12].

To ensure patients’ safety, pharmacists played an essential role in screening and evaluating the clinical history of patients before the initiation of Paxlovid^®^ [10]. Pharmacists were tasked to identify patients with contraindications, such as those less than 18 years old, with symptoms onset more than 5 days, patients who required oxygen, with severe hepatic impairment (Child–Pugh Class C), severe kidney impairment (eGFR < 30 ml/min), pregnancy or breastfeeding. For patients with moderate renal impairment (eGFR 30–60 ml/min), renal adjustment dosage was recommended. Furthermore, pharmacists screened for hypersensitivity to the Paxlovid^®^ active ingredients or excipients, contraindicated medications, as well as other drug interactions based on information on the Infectious Diseases Society of America COVID-19 treatment and management guideline [13] and online drug interaction checker [14].

Pharmacists also act as an intermediary to facilitate communication between prescribers and patients [15]. This was crucial as Paxlovid^®^ was newly introduced and not prescribed routinely. In this case, patients can exert an informed decision after counselling by the pharmacists. They can choose whether to take Paxlovid^®^. Patients were informed by the pharmacists that they were suitable candidates, and their decision would be communicated by the pharmacists to the prescribers [15]. Patients who rejected Paxlovid^®^ were reviewed by the prescribers. Prescribers then decide whether to omit the treatment or provide an alternative based on the risk–benefit ratio, i.e., judging on whether the patient will progress to a more severe COVID-19 stage.

### Dispensing

Before dispensing to the patients, a pharmacist’s core responsibility is to assure that the prescriptions are accurate so that patients receive the correct medication and the appropriate dosage [10]. At this point, pharmacists will assess the appropriateness, efficacy, and safety of Paxlovid^®^ for each patient. Using the “five rights” practise [16] necessitates a pharmacist extensively reviewing each patient’s medication list, age, weight, ethnicity, diet, allergies, and kidney and liver function, which may result in suggestions for modifications in therapy or monitoring to ensure medication safety.

Patients receiving ambulatory care were dispensed ten doses of Paxlovid^®^ at once (full 5-day treatment course). Following that, the pharmacists convey clear instructions on how to take the medications supplied. For patients who were warded and prescribed with Paxlovid^®^, clinical pharmacists were tasked with supplying Paxlovid^®^ on a daily unit dose basis and provide drug-related information to clinical staff, including side effects, drug–drug interactions, and drug–food interactions [17]. Clinical pharmacists were required to obtain a complete history of all medications, including over-the-counter and traditional medications and review patients’ COVID-19 clinical staging, excluding severe or critical patients, before supplying medications to the medical team [10].

### Counselling

Pharmacists play an important role in educating patients regarding the instructions for drug use, drug storage, and the side effects that they need to take note of. Like other antiviral therapies, non-adherence to Paxlovid^®^ may increase the risk of drug resistance and cause treatment failure [18]. Involvement of pharmacists in patient counselling during discharge and follow-up was reported to improve adherence and patients’ satisfaction [19]. In our setting, pharmacists were responsible for convincing and reassuring patients regarding the safety of Paxlovid^®^ and the importance of completing the treatment. Patients were also counselled not to share Paxlovid^®^ with other family members who contracted COVID-19.

The roles and responsibilities of pharmacists in counselling have expanded greatly during the COVID-19 pandemic [20]. In our setting, pharmacists counselled patients regarding Paxlovid^®^ indication, the mode of action, the method of administration, the contraindications and drug interactions, actions to take when missing a dose or overdose happens, side effects, storage and disposal methods, as well as mechanism of reporting any adverse drug reaction [21]. Patients were warned regarding the safety of the drug in pregnancy and breastfeeding, and emphasized that contraception must be practiced 7 days after taking Paxlovid^®^ [21]. While we did not quantify the impact of the pharmacist-led counselling initiatives in reducing Paxlovid^®^-related adverse drug events, a previous study with a similar approach demonstrated that pharmacist counselling at discharge supplemented with 3rd and 5th day follow-up phone call significantly reduced preventable adverse drug events 30 days post-discharge [22].

### Monitoring and reporting

Pharmacovigilance, or the monitoring of adverse drug reactions, is critical in ensuring patients’ safety, especially in the case that the therapeutic agent is novel with limited safety data. The cost of managing ADR was exorbitant, reported at more than USD 10,000 per ADR [23]. In early October 2022, the Malaysian National Pharmaceutical Regulatory Agency received 1001 reports with 1714 adverse events related to Paxlovid^®^. The most reported adverse events were bitter taste (562 cases), diarrhoea (247 cases), dysgeusia (134 cases), vomiting (70 cases) and nausea (69 cases) [24]. Timely detection of Paxlovid^®^-related ADR could reduce the health system’s financial burden and improve the patient’s outcome. Apart from soliciting common ADR, pharmacists also actively monitor uncommon ADR, including anaphylaxis and hypersensitivity reactions [25].

In our setting, the pharmacists were required to follow-up all patients started on Paxlovid^®^ via phone call on Day 3 and Day 5 post-initiation, examining both adherence and ADR. To improve call response, patients were informed before discharge or during their last encounter that they would be contacted by pharmacists on Day 3 and Day 5 following Paxlovid^®^ initiation.

The schedule was rationalized as follow-up patients after the 1st week of discharge could be beneficial in reducing readmissions [26]. Pharmacists recorded Paxlovid^®^ treatment adherence and ADR into the existing patient administration registry based on patients’ self-reports. In non-adherence cases, pharmacists explored the reasons and provided additional counselling. If patients insisted on stopping Paxlovid^®^ treatment, the pharmacist advised them to return the medication to the nearest pharmacy unit.

Pharmacists are also responsible for reporting Paxlovid^®^-related adverse drug reactions to a central database hosted by the National Pharmaceutical Regulatory Agency, through either the pharmacy information system, online web form, or manual submission [27]. Patients were advised by pharmacists to seek medical assistance at the nearest health clinic if they experienced Paxlovid^®^-related ADR, especially if the event was severe or unusual.

### Challenges

**Fig. 1:**
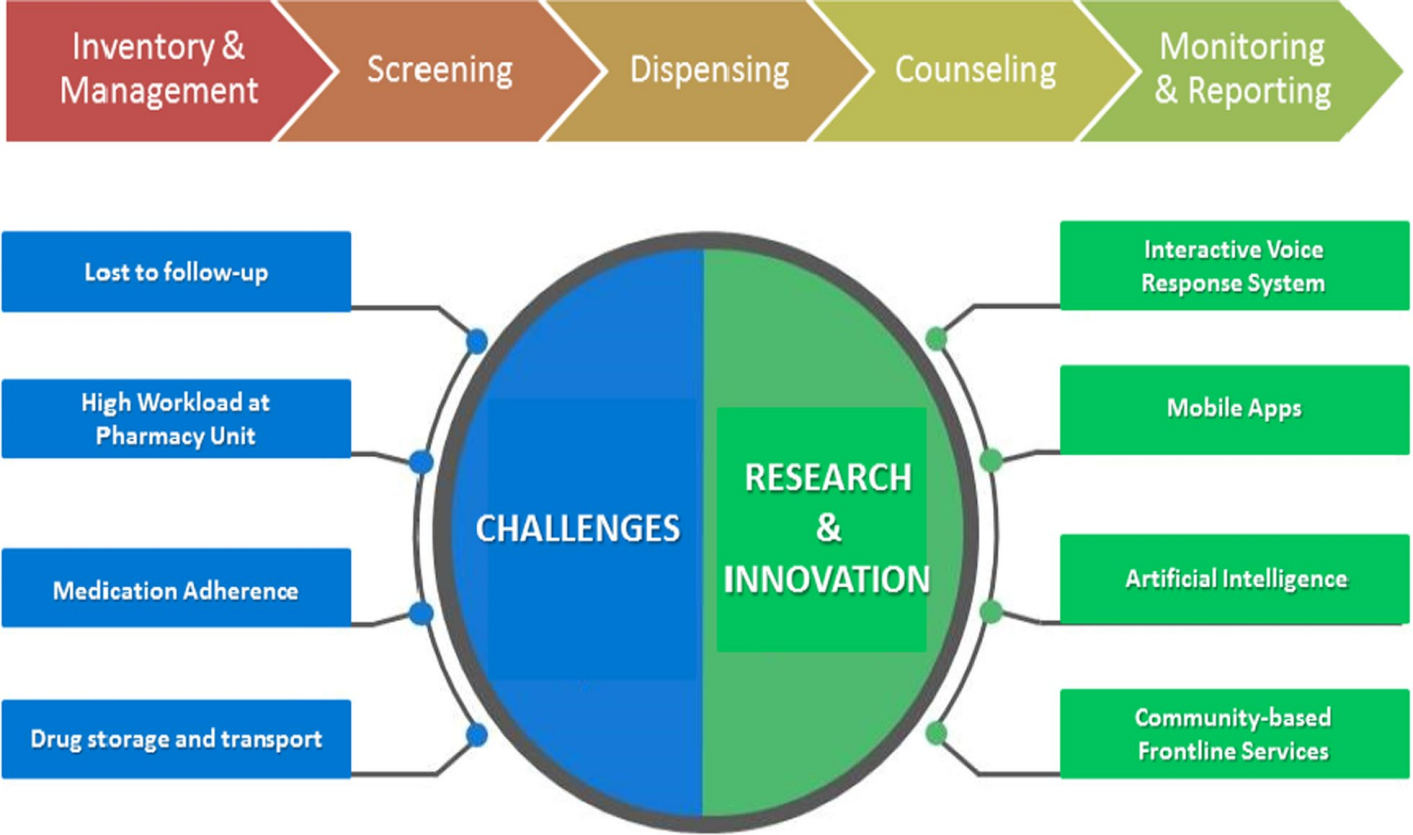
Role of pharmacists, challenges and research opportunities in managing novel drugs

We experienced several challenges in managing Paxlovid^®^, including follow-up, workload, medication storage, and adherence (Fig. 1). One of the major obstacles faced by pharmacists in performing follow-up was the limitation of the manual phone call method. Calling patients manually entails a high workload, involving a team of designated pharmacists. Each Paxlovid^®^ follow-up phone call lasted about 10 min and each patient required three follow-ups. This was compounded by the pharmacists’ existing workloads, who were expected to meet the patient waiting time target as well as complete other clinical and administrative duties. At the time of writing, there were more than 650 patients started with Paxlovid^®^ in Perak state.

Moreover, unanswered phone calls complicate the follow-up efforts. Some patients did not pick up calls after discharge, whilst some provided invalid phone numbers. Based on the local protocol, pharmacists were required to call the same number at least three times before excluding the patient from Paxlovid^®^ phone call follow-up. The traditional phone call follow-up method entailed a high drop-out. In the United States, almost half of the patients could not be reached by phone call after discharge [28]. Furthermore, a minority of underprivileged population from lower socio-economical backgrounds did not own a phone, posing a different challenge in Paxlovid^®^ phone call follow-up.

Besides, stringent storage and transport requirements must be met to ensure the stability of Paxlovid^®^. According to the FDA [9], Paxlovid^®^ must be stored at a controlled room temperature between 20 and 25 °C. Any excursion below 15 °C and exceeding 30 °C may cause the drug to no longer be usable. Pharmacists were required to monitor the temperature of Paxlovid^®^ closely throughout the transit, storage, and dispensing process.

Ensuring adherence to Paxlovid^®^ therapy was another challenge faced by pharmacists. Some patients deliberately stopped their medication, rationalizing that their symptoms had improved over time. While pharmacists strived to explain the importance of treatment adherence, this could not be achieved in every patient. From a clinical point of view, this may compromise the efficacy of Paxlovid^®^ therapy. From the economic perspective, the omission of doses causes wastage, as the average cost of one complete Paxlovid^®^ regimen was estimated at USD 250 [29].

### Research and innovation

Innovative measures could be considered to overcome administrative, technical, and clinical challenges (Fig. 1). In the face of the COVID-19 pandemic, virtual patient follow-up via phone and video calls was crucial to enhance patients’ access to healthcare [30]. However, the manual phone call follow-up to monitor Paxlovid^®^ adverse events was resource intensive. A previous study found that calling patients using an Interactive Voice Response System was not inferior to human-manual calls [31]. This could largely reduce the pharmacist’s burden, provided that the system is mature enough to be distinguished from a spam call. Meanwhile, as some patients might feel intruded by a telephone call, alternative communication methods such as email and texting could be offered at the point of discharge to improve the follow-up response rate [32].

The Malaysian public hospitals serve a diverse population, including hard-to-reach and marginalized people, who have not responded positively to our Paxlovid^®^ follow-up phone call. Active engagement of pharmacists through network and relationship building with key stakeholders such as the Department of Orang Asli Development (JAKOA), existing community organizations, and health volunteers could be instrumental in designing effective and culturally sensitive Paxlovid^®^ follow-up programmes to improve access for this population [33–35].

Pharmacists play an instrumental role in optimizing patients’ adherence to Paxlovid^®^. Timely review and integration of adherence-based mobile application into medication counselling serves as an important measure to improve Paxlovid^®^ adherence [36]. Preliminary evidence suggests that mobile applications may improve patients’ adherence to medications [37]. Integration of the Paxlovid^®^ adherence monitoring function into the widely used MySejahtera application could be one cost-effective option [38]. Moving forward, the feasibility and clinical impact of integrating artificial intelligence into these mobile applications should be explored [39].

Traditionally, ADRs are reported by healthcare professionals, including pharmacists. However, underreporting of ADR was common due to administrative and technical barriers [23]. Implementation of an electronic ADR reporting system could be instrumental in facilitating and promoting Paxlovid^®^-related ADR reporting, especially when such a system is integrated into the hospital information system [40]. Furthermore, the public should be educated on ADR reporting mechanisms to reduce ADR reporting workloads among health professionals. To facilitate reporting, a user-friendly interface should be introduced, for example by integrating such a function into the MySejahtera application.

## Conclusion

Pharmacists are holding the ground firmly during the COVID-19 pandemic. The discovery of novel treatment agents such as Paxlovid^®^ holds pharmacists accountable for inventory management, screening, dispensing, counselling, monitoring, and reporting, among others. Wearing multiple hats, a pharmacist’s involvement in research is critical to innovative communication and pharmacovigilance systems. Advancements in technologies warrant digitalization in pharmacy services. Transformation could begin by shifting from manual follow-up and reporting to electronic and automation models, encompassing artificial intelligence in the near future.

## Data Availability

The datasets used in the current study are not publicly available, but are available from the corresponding author on reasonable request.

## References

[1] Wang Q, Guo Y, Iketani S, Nair MS, Li Z, Mohri H (2022). Antibody evasion by SARS-CoV-2 Omicron subvariants BA.2.12.1, BA.4, & BA.5. Nature.

[2] Hammond J, Leister-Tebbe H, Gardner A, Abreu P, Bao W, Wisemandle W (2022). Oral nirmatrelvir for high-risk, nonhospitalized adults with Covid-19. N Engl J Med.

[3] Visacri MB, Figueiredo IV, Lima TdM (2021). Role of pharmacist during the COVID-19 pandemic: a scoping review. Res Soc Adm Pharm.

[4] Elbeddini A, Prabaharan T, Almasalkhi S, Tran C (2020). Pharmacists and COVID-19. J Pharm Policy Pract.

[5] Thong KS, Selvaratanam M, Tan CP, Cheah MF, Oh HL, Lee PM (2021). Pharmacy preparedness in handling COVID-19 pandemic: a sharing experience from a Malaysian tertiary hospital. J Pharm Policy Pract.

[6] Bukhari N, Rasheed H, Nayyer B, Babar ZUD (2020). Pharmacists at the frontline beating the COVID-19 pandemic. J Pharm Policy Pract.

[7] Ammar MA, Tran LJ, McGill B, Ammar AA, Huynh P, Amin N (2021). Pharmacists leadership in a medication shortage response: illustrative examples from a health system response to the COVID-19 crisis. J Am Coll Clin Pharm.

[8] Ministry of Health Malaysia. Garis Panduan Peluasan Penggunaan Rawatan Ubat Antiviral Covid-19 Bagi Fasiliti/Pengamal Perubatan Swasta. 2022. p. 28. https://covid-19.moh.gov.my/garis-panduan/garis-panduan-kkm/ANNEX-2r-Garispanduan-Peluasan-Paxlovid-ke-Fasiliti-Swasta-25072022.pdf. Accessed 8 Sept 2022.

[9] Fact sheet for healthcare providers: emergency use authorization for paxlovid. 2022. p. 35. https://www.fda.gov/media/155050/download. Accessed 8 Sept 2022.

[10] Song Z, Hu Y, Zheng S, Yang L, Zhao R (2021). Hospital pharmacists’ pharmaceutical care for hospitalized patients with COVID-19: recommendations and guidance from clinical experience. Res Soc Adm Pharm.

[11] Ministry of Health Malaysia. Clinical management of confirmed covid-19 case in adult and paediatric. 2022. p. 29. https://covid-19.moh.gov.my/garis-panduan/garis-panduan-kkm/ANNEX-2E-CLINICAL-MANAGEMENT-OF-CONFIRMED-COVID-19-31052022.pdf. Accessed 8 Sept 2022.

[12] Prioritization of therapeutics. COVID-19 treatment guidelines. 2022. https://www.covid19treatmentguidelines.nih.gov/overview/prioritization-of-therapeutics/. Accessed 11 Oct 2022.

[13] Infectious Diseases Society of America. Management of drug interactions with nirmatrelvir/ritonavir (Paxlovid^®^): resource for clinicians. 2022. https://www.idsociety.org/practice-guideline/covid-19-guideline-treatment-and-management/management-of-drug-interactions-with-nirmatrelvirritonavir-paxlovid. Accessed 9 Aug 2022.

[14] University of Liverpool. COVID-19 drug interactions. https://www.covid19-druginteractions.org/checker. Accessed 9 Aug 2022.

[15] Tanne JH (2022). Covid-19: FDA authorises pharmacists to prescribe Paxlovid. BMJ.

[16] Grissinger M (2010). The five rights: a destination without a map. Pharm Ther.

[17] Abbasi Nazari M, Salamzadeh J, Hajebi G, Gilbert B (2011). The role of clinical pharmacists in educating nurses to reduce drug-food interactions (absorption phase) in hospitalized patients. Iran J Pharm Res.

[18] Bezabhe WM, Chalmers L, Bereznicki LR, Peterson GM (2016). Adherence to antiretroviral therapy and virologic failure: a meta-analysis. Medicine.

[19] Sanii Y, Torkamandi H, Gholami K, Hadavand N, Javadi M (2016). Role of pharmacist counseling in pharmacotherapy quality improvement. J Res Pharm Pract.

[20] Bragazzi N, Mansour M, Bonsignore A, Ciliberti R (2020). The role of hospital and community pharmacists in the management of COVID-19: towards an expanded definition of the roles, responsibilities, and duties of the pharmacist. Pharmacy.

[21] Ritonavir-Boosted Nirmatrelvir (Paxlovid). COVID-19 treatment guidelines. 2022. https://www.covid19treatmentguidelines.nih.gov/therapies/antiviral-therapy/ritonavir-boosted-nirmatrelvir--paxlovid-/. Accessed 11 Oct 2022.

[22] Schnipper JL, Kirwin JL, Cotugno MC, Wahlstrom SA, Brown BA, Tarvin E (2006). Role of pharmacist counseling in preventing adverse drug events after hospitalization. Arch Intern Med.

[23] Hadi MA, Neoh CF, Zin RM, Elrggal M, Cheema E (2017). Pharmacovigilance: pharmacists’ perspective on spontaneous adverse drug reaction reporting. IPRP.

[24] Mei CS. Nirmatrelvir/Ritonavir (PAXLOVID): risk of anaphylaxis and hypersensitivity reactions. 2022. https://www.npra.gov.my/index.php/en/health-professionals/recent-updates/435-english/safety-alerts-main/safety-alerts-2022/1527403-nirmatrelvir-ritonavir-paxlovidtm-risk-of-anaphylaxis-and-hypersensitivity.html. Accessed 11 Oct 2022.

[25] European medicines Agency. Paxlovid for the treatment of Covid-19. 2021. p. 131. https://www.ema.europa.eu/en/documents/referral/paxlovid-pf-07321332-ritonavir-covid-19-article-53-procedure-assessment-report_en.pdf. Accessed 8 Sept 2022.

[26] Lee KK, Yang J, Hernandez AF, Steimle AE, Go AS (2016). Post-discharge follow-up characteristics associated with 30-day readmission after heart failure hospitalization. Med Care.

[27] National Pharmaceutical Regulatory Agency. Adverse Drug Reaction (ADR)/Adverse Event Following Immunisation (AEFI) reporting. 2021. p. 41. https://www.npra.gov.my/easyarticles/images/users/1047/Adverse-Drug-Reaction-ADR--Adverse-Event-Following-Immunisation-AEFI-Reporting-Manual-For-Healthcare-Providers.pdf. Accessed 8 Sept 2022.

[28] Adams SL, Thompson DA (1996). Inability to follow up ED patients by telephone: there must be 50 ways to leave your number. Acad Emerg Med.

[29] Ministry of Health Malaysia. Protease inhibitors (paxlovid^®^)—oral treatment for covid-19. 2021. p. 4. https://covid-19.moh.gov.my/kajian-dan-penyelidikan/mahtas-covid-19-rapid-evidence-updates/Rapid-Review-Paxlovid-Oral-treatment-for-covid-19-04022022.pdf. Accessed 8 Sept 2022.

[30] Elbeddini A, Yeats A (2020). Pharmacist intervention amid the coronavirus disease 2019 (COVID-19) pandemic: from direct patient care to telemedicine. J Pharm Policy Pract.

[31] Houser SH, Ray MN, Maisiak R, Panjamapirom A, Willig J, Schiff GD (2013). Telephone follow-up in primary care: can interactive voice response calls work?. Stud Health Technol Inform.

[32] Agency for Healthcare Research and Quality. Tool 5: how to conduct a postdischarge follow-up phone call. 2013. https://www.ahrq.gov/patient-safety/settings/hospital/red/toolkit/redtool5.html. Accessed 9 Aug 2022.

[33] Sevelius JM, Gutierrez-Mock L, Zamudio-Haas S, McCree B, Ngo A, Jackson A (2020). Research with marginalized communities: challenges to continuity during the COVID-19 pandemic. AIDS Behav.

[34] Nungsari M, Yin CH, Fong N, Pillai V. Understanding the impact of the COVID-19 outbreak on vulnerable populations in Malaysia through an ethical lens: a study of non-state actors involved in aid distribution. Wellcome Open Research; 2022. https://wellcomeopenresearch.org/articles/6-263. Accessed 11 Oct 2022. 10.12688/wellcomeopenres.17239.1 PMC8785141 35111977

[35] Chung MHL, Hazmi H, Cheah WL (2017). Role performance of community health volunteers and its associated factors in Kuching District, Sarawak. J Environ Public Health.

[36] Haase J, Farris KB, Dorsch MP (2017). Mobile applications to improve medication adherence. Telemed e-Health.

[37] Palmer MJ, Barnard S, Perel P, Free C (2017). Mobile phone-based interventions for improving adherence to medication prescribed for the primary prevention of cardiovascular disease in adults. Cochrane Database Syst Rev.

[38] John Leon Singh H, Couch D, Yap K (2020). Mobile health apps that help with COVID-19 management: scoping review. JMIR Nurs.

[39] Tursunbayeva A, Renkema M (2022). Artificial intelligence in health-care: implications for the job design of healthcare professionals. Asia Pac J Hum Res.

[40] Ortega A, Aguinagalde A, Lacasa C, Aquerreta I, Fernández-Benítez M, Fernández LM (2008). Efficacy of an adverse drug reaction electronic reporting system integrated into a hospital information system. Ann Pharmacother.

